# Application of ultrasound to enhance the silt drying process: An experimental study

**DOI:** 10.1371/journal.pone.0236492

**Published:** 2020-07-31

**Authors:** Jianxiang Guo, Guohui Lei

**Affiliations:** 1 Key Laboratory of Ministry of Education for Geomechanics and Embankment Engineering, Hohai University, Nanjing, China; 2 Key Laboratory of Ministry of Education for Geomechanics and Embankment Engineering, Hohai University, Nanjing, China; University of Vigo, SPAIN

## Abstract

Scientific and reasonable treatment of dredged silt can not only protect the ecological environment but also play an essential role in the utilization of silt resources. Due to high water content, low permeability and high organic matter content of the silt, a large amount of bacteria and harmful gases are often produced during the process of silt sedimentation. Thermal drying has been taken as a technically attractive method for harmless treatment of contaminated dredged silt. In this study, ultrasound technology is introduced to shorten the time needed for silt drying. A preliminary laboratory study is carried out to assess the effectiveness of ultrasound on thermal drying. A series of thermal drying tests, with and without ultrasound, were conducted on kaolin soil specimens that were prepared by settling and self-weight consolidation. The test results show that the length of drying time can be shortened by increasing temperature and ultrasound power. The drying time plays a dominant role in the determination of the total energy consumption. This is because reduction of drying time leads to significant decrease in energy consumption for thermal drying, and the energy consumption for additional ultrasound is relatively marginal. For thermal drying at temperatures 60 and 100°C, when combined with 100 W ultrasound, the length of drying time was shortened by 44.19% and 45.16%, and the energy consumption was saved by 30.07% and 38.16%, respectively; when combined with 60 W ultrasound, the length of drying time was shortened by 4.65% and 6.45%, but the energy consumption was increased by 9.79% and 0.48%, respectively. The combination of thermal drying and 100 W ultrasound is found to be optimal in terms of drying rate and energy consumption for silt drying.

## Introduction

There are many navigable rivers around the world. A large amount of silt deposits on the riverbeds due to sedimentation every year. Dredging is a common method used to maintain the navigable capacity of rivers. In this method, silt is sucked out from riverbeds and pumped into an open area for natural settling and artificial treatment, during which the organic compounds contained in the dredged silt generate and release unpleasant odors and cause pollution [1]. The dredged silt is taken as waste until it can be transported without being easily leaked/spilled out of containers, and utilized as a valuable material without pollution [2]. It is difficult for pore liquid of silt to migrate outward [3–5], and the natural settling process without the aid of artificial treatment may last for years. This is because the initial water content of dredged silt is exceptionally high, yet the permeability of the dredged silt is very poor. Therefore, conducting a harmless and efficient dehydration treatment is a necessary step to reuse the silt for engineering or agricultural purposes and avoid the negative impact on the environment [6].

The thermal drying technology, which is widely used in chemical industry, is considered a time and cost–effective method for harmless dehydration treatment of dredged silt. The treatment process includes two parts: surface water evaporation and internal water diffusion [7]. Internal water diffusion is the dominant part affecting the rate of drying.

Some researchers have applied ultrasound technology to accelerate the evaporation of moisture [8–12]. Focuses have been placed on the effects of ultrasound on destructing soil structure, promoting drainage of pore water, and accelerating consolidation of soil [9–12]. Kim et al. [9] observed that ultrasound promotes osmotic dehydration of soil. When the pores between soil particles are filled with water, sound waves are absorbed and converted to heat, and the cavitation caused by ultrasound also helps to remove strong bound water [10, 11]. Riera-Franco de Sarabia et al. [12] found that ultrasound produces a spongy effect in pores, causing a rapid series of alternative compressions and expansions, and promoting the passage of water through pore channels. In these cases, water content was reduced by the action of ultrasound.

Given the significant effects of both heat and ultrasound on drying, some scholars have combined thermal drying with ultrasound technology. Hot air drying of coal assisted by ultrasound has received scholarly attention and analyzed in detail for its potential benefits [13–14]. Thermal drying combined with ultrasound has also been used to shorten the drying time in vegetable (up to 18–27%) [15] and fruit (up to 31%) [16].

**Fig 1:**
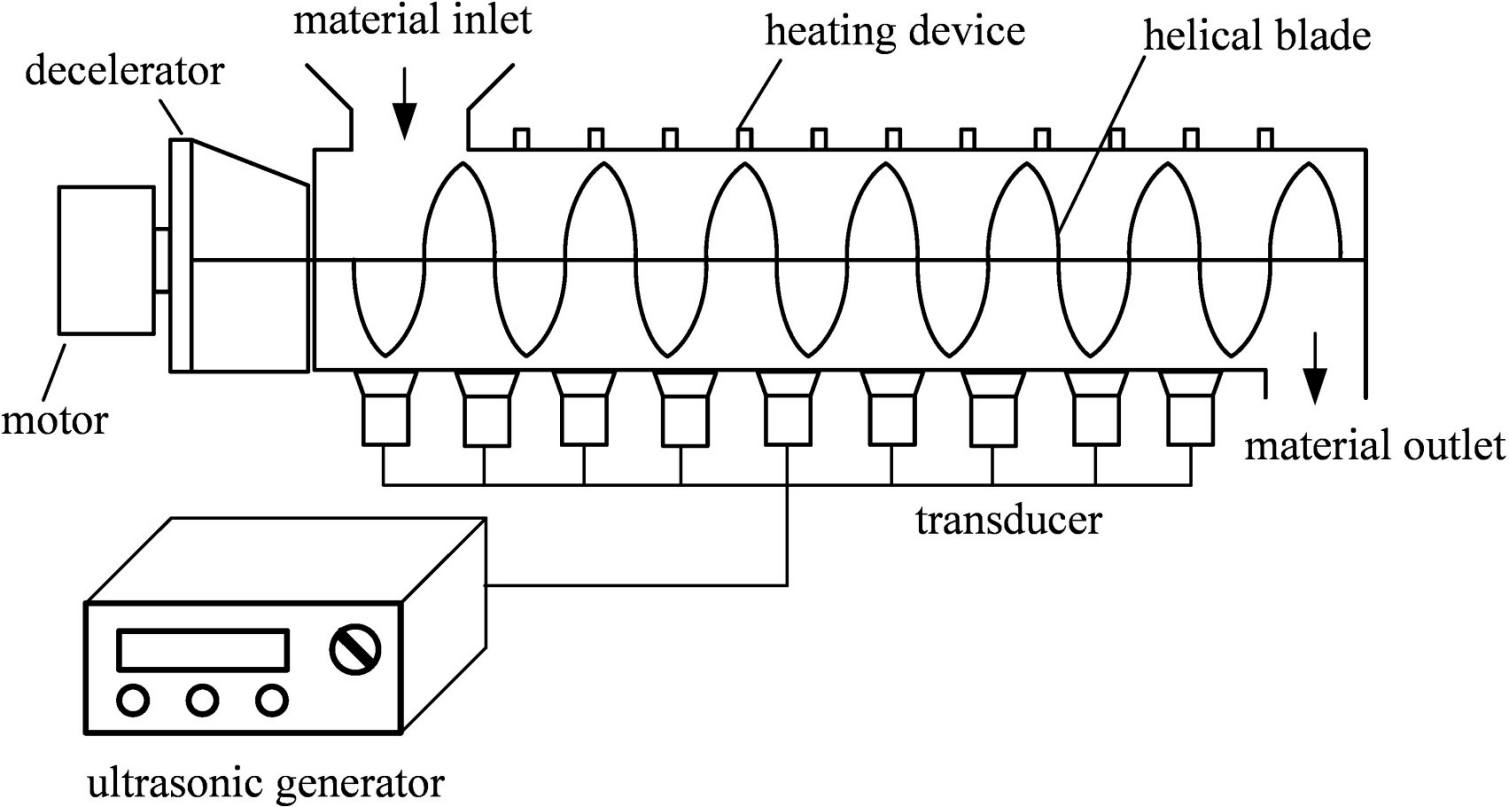
Schematic of an equipment for thermal drying combined with ultrasound.

Some scholars have developed large-scale equipment for drying industrial products using a combination of heat and ultrasound [12, 17, 18]. Some large-scale ultrasound equipment has also been used to remove organic matter from dredged silt [19]. For harmless dehydration treatment of dredged silt, a large-scale heating equipment equipped with ultrasonic facility may be designed, as schematically shown in Fig 1.

It should be noted that the thermal, cavitation and mechanical effects of the ultrasound itself work differently on various materials [9, 20–22]. To investigate the effect of ultrasound on dredged soil drying process, a series of laboratory experiments were carried out in this study. The effects of temperature, ultrasound power and frequency on water loss were examined. The combination (ultrasound and thermal drying) was optimized by searching for the operating scheme that can minimize the total drying time.

This paper is organized as follows. First, the test materials and methods are presented. Then the results are discussed, followed by the summary and conclusions.

## Materials and methods

### Material

**Fig 2:**
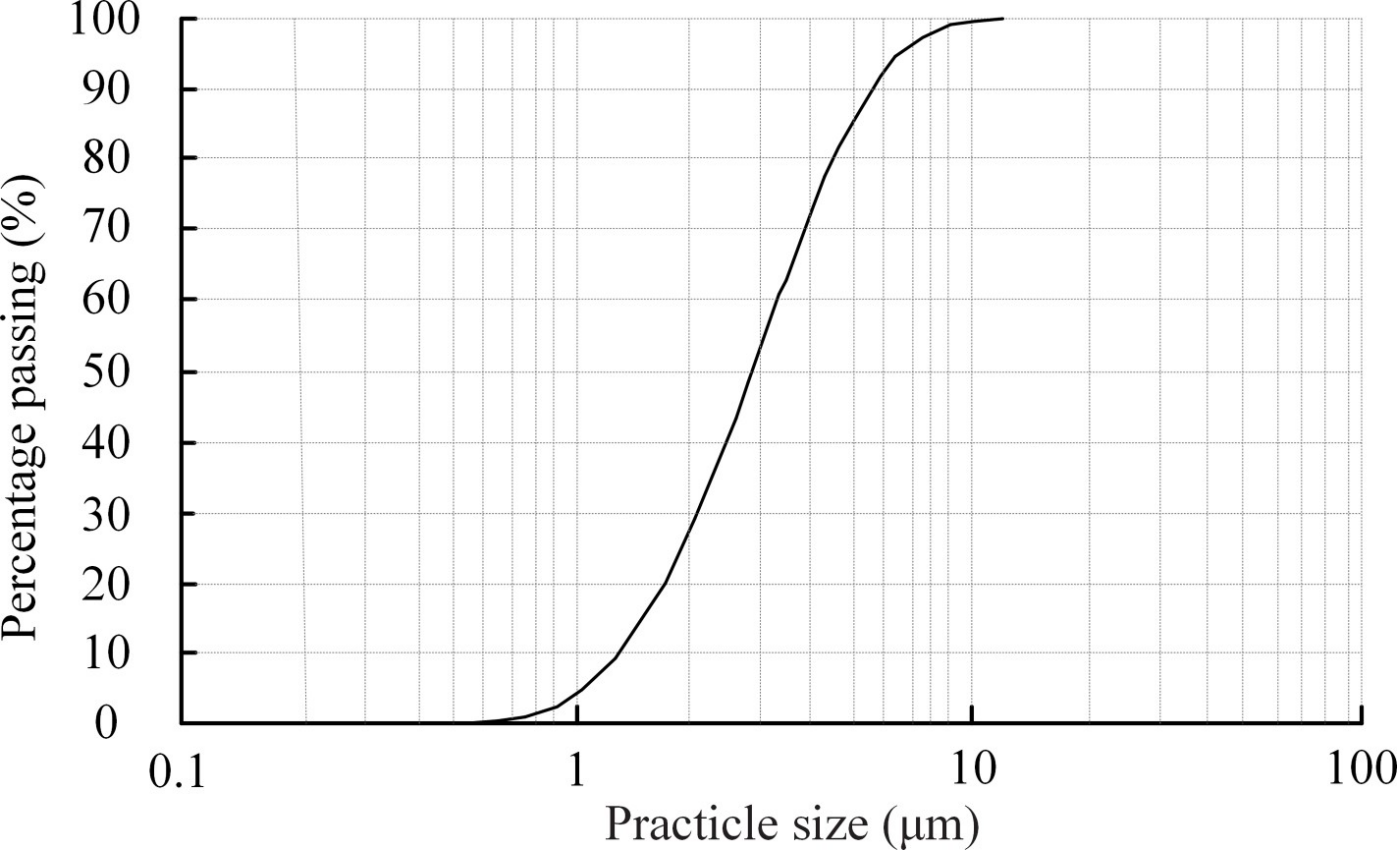
Particle size distribution curve of the kaolin.

Kaolin was used in this study. The particle size distribution curve of the kaolin was obtained using a laser particle–size analyzer, as shown in Fig 2. It shows that the particle size of the kaolin ranges from 0.6 μm to 10 μm.

### Experimental setup

**Fig 3:**
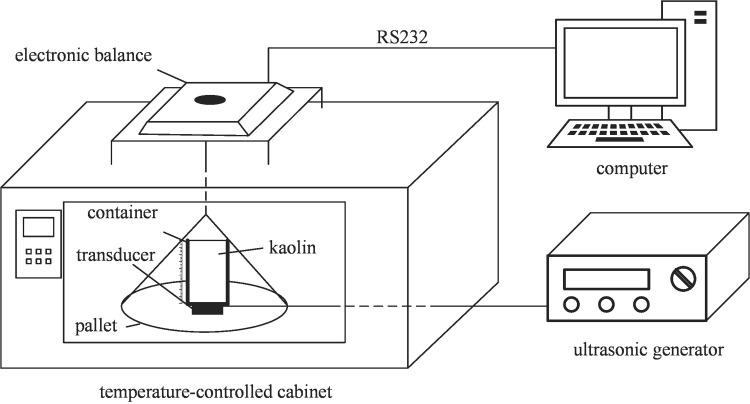
Schematic of the experimental setup.

Fig 3 shows a schematic of the experimental setup used in this study. It consists of a temperature-controlled cabinet, a soil specimen container, an electronic balance, an ultrasound transducer, an ultrasound generator and a data acquisition computer. Ultrasound was generated by a tunable power ultrasonicate equipped with transducers of various frequencies. The soil specimen container was made of plexiglass with an inner diameter of 50 mm and a height of 100 mm. A graduated ruler was mounted on the outer surface of the container. A transducer was firmly attached to the bottom of the container by AB glue. The ultrasound equipment kept operating during the drying process. The water loss in the soil specimen was measured by the electronic balance, and the measured data was transmitted to the computer. The temperature-controlled cabinet can provide a high temperature up to 300°C.

### Specimen preparation

Soil specimens were prepared by the sedimentation method, which is similar to the formation process of silt underwater. First, kaolin was purified by eliminating the impurities with a 1000 mesh sieve and put into an oven for 24 hours at a temperature of 105°C to remove the moisture. Then, the amount of required kaolin was weighed, carefully placed into the specimen container, and mixed with distilled water to form a dilute slurry at a water content more than twice the liquid limit of the kaolin [23]. Careful removal of the surface water was conducted after sedimentation lasting for 3 days, then the subsequent test was carried out. During the test, the mass of the soil specimen was measured by the electronic balance and recorded by computer. After the test, the specimen was put into an oven for 24 hours at 105°C. The total mass of water contained in the specimen was the difference between the masses of the soil specimen measured before and after the specimen was oven dried. Table 1 shows the initial data of the specimens tested after self-weight settling and consolidation for three days, the corresponding temperature and ultrasound parameters adopted in each test. The initial water content of the specimen is defined as the ratio of the initial mass of water to the mass of soil particles in the specimen.

**Table 1:** Initial data for each test.

Identity	Initial water content /%	Specimen thickness /mm	Temperature /°C	Ultrasound parameters
1	65.87	57.5	–	100 W 20 kHz
2	64.29	57.3		100 W 28 kHz
3	65.09	57.4		100 W 40 kHz
4	64.01	57.3	60	–
5	65.24	57.4		60 W 40 kHz
6	65	57.4		100 W 20 kHz
7	64.89	57.4		100 W 28 kHz
8	64.23	57.3		100 W 40 kHz
9	63.88	57.2	70	–
10	64.36	57.4		60 W 40 kHz
11	64.22	57.3		100 W 40 kHz
12	63.2	57.2	80	–
13	64.33	57.3		60 W 40 kHz
14	`64.76	57.5		100 W 40 kHz
15	64.56	57.3	90	–
16	64.9	57.5		60 W 40 kHz
17	65.14	57.6		100 W 40 kHz
18	64.34	57.2	100	–
19	63.54	57.1		60 W 40 kHz
20	64.7	57.4		100 W 40 kHz

### Test procedure

For comparison purposes, two kinds of drying modes were performed. One was the thermal drying at different temperatures (i.e., 60, 70, 80, 90 and 100°C), and the other was the thermal drying combined with ultrasound at different acoustic frequencies (i.e., 20, 28 and 40 kHz) and power levels (i.e., 60 and 100 W). The environmental air temperature and relative humidity of the laboratory were maintained as constant as possible throughout all the experiments, and they were kept as 20 ± 2°C and 50 ± 5%, respectively. The mass of water contained in each soil specimen changed during the experiments. The change in mass of water can be derived from the difference in the total mass of the specimen, pallet, transducer and container (see Fig 3), which was recorded automatically by the electronic balance every five minutes. The ultimate mass of water, which corresponds to the end of the drying process, was determined when the variation in the total mass became negligible.

The drying behavior of the soil specimens was analyzed based on the corresponding drying curves. The drying curve shows the water loss of the soil with time. The drying curve is plotted using time *t* as the abscissa and moisture ratio *MR* as the ordinate. The influence of different factors on drying was analyzed by comparing the drying curves under different test conditions. The moisture ratio is defined as follows [24]:

$$MR=\frac{M_{\text{t}}-M_{\text{e}}}{M_{0}-M_{\text{e}}}\times 100\text{\%}$$ (1)

where *M*_t_ is the mass of water in the soil specimen at a point in time *t*; *M*_0_ is the initial value of the mass of water in the saturated soil specimen; *M*_e_ is the mass of water remained in the soil specimen at the end of the drying process. It can be seen from Eq (1) that *MR* eliminates the influence caused by different initial water contents and also provides convenience for the analysis of the experimental data and the drying behavior.

### Statistical analysis

To study the effects of temperatures and ultrasound parameters on the drying time and energy consumption, an analysis of variance (ANOVA) was carried out using Microsoft Excel^®^ 2019. Statistically significant differences were calculated using Tukey’s test [25]. The results of Tukey’s test include the sum of squares (SS), degrees of freedom (DF), mean square (MS), *F* value and *p* value, as detailed in [26]. The *F* value is given by dividing the absolute value of the difference between pairs of means by the standard error of the means. It is used to determine whether the effect is significant. The *p* value is determined by the *F* statistic. It represents the probability that the results could have happened by chance, and *p* < 0.05 is normally considered statistically significant.

## Results

### Thermal drying without ultrasound

**Fig 4:**
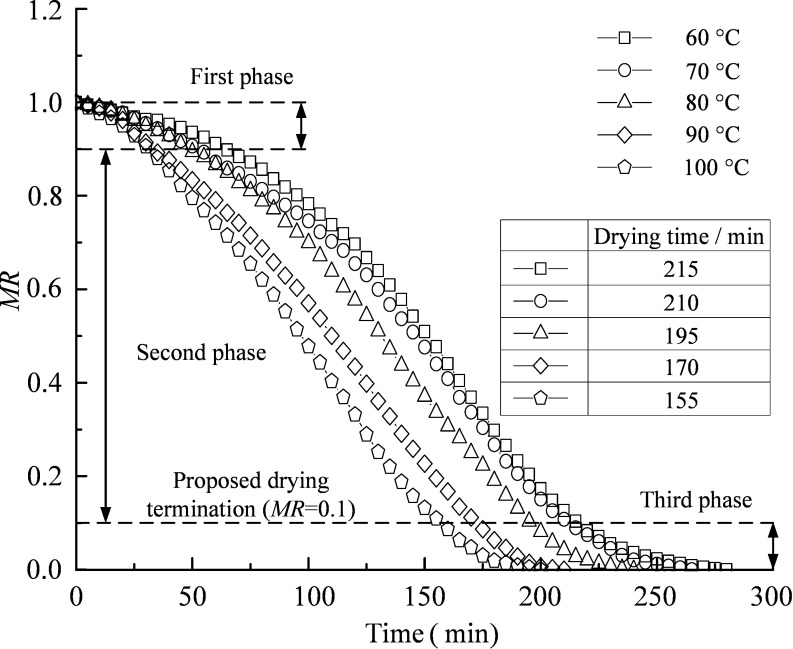
Variations in moisture ratio with time under different temperatures.

Fig 4 shows the effect of temperature (60–100°C) on the moisture ratio of the soil. The slope of the drying curve represents the rate of drying. In Fig 4, the drying rate increases with the increase in temperature. This is because heating reduces the dynamic viscosity of pore water, promotes the migration of pore water in soil skeleton, and facilitates the evaporation of water from the soil surface. Similar behavior has also been observed in the drying tests of strawberries [27].

In Fig 4, there are three phases during every drying process. In the first phase (*MR* from 1 to 0.9), the internal energy of the specimen increases, and the surface water evaporates. The drying rate increases gradually, thus the drying curve behaves as a downward bending shape. In this phase, the drying rate mainly depends on the absorption of the energy in the specimens. In the second phase (*MR* from 0.9 to 0.1), the moisture evaporation tends to be stable, and the drying rate is dependent on the diffusion of the internal moisture. The drying rate remains approximately constant, and the drying curve becomes an approximately straight line. In the third phase (*MR* from 0.1 to 0), the water in the silt mainly consisted of residual capillary water and bound water. Compared with pore water, capillary water and bound water have stronger binding force and migration resistance. Hence, the internal water diffusion slows down, and the drying rate begins to decrease to zero gradually. The drying rate in the third phase is the lowest, and the lengths of time for *MR* changing from 0.1 to 0 at five different temperatures (i.e. 60°C, 70°C, 80°C, 90°C and 100°C) are 23.21%, 20.75%, 18.75%, 19.05% and 22.5% of their total lengths of drying time, respectively. However, complete drying is time and cost consuming and not necessary for engineering purpose. So, *MR* = 0.1 is proposed as a proxy for evaluating the drying time needed for practical termination of silt drying. By increasing the temperature from 60 to 100°C, the length of drying time is shortened by 27.91%.

### Effect of ultrasound frequency on drying

**Fig 5:**
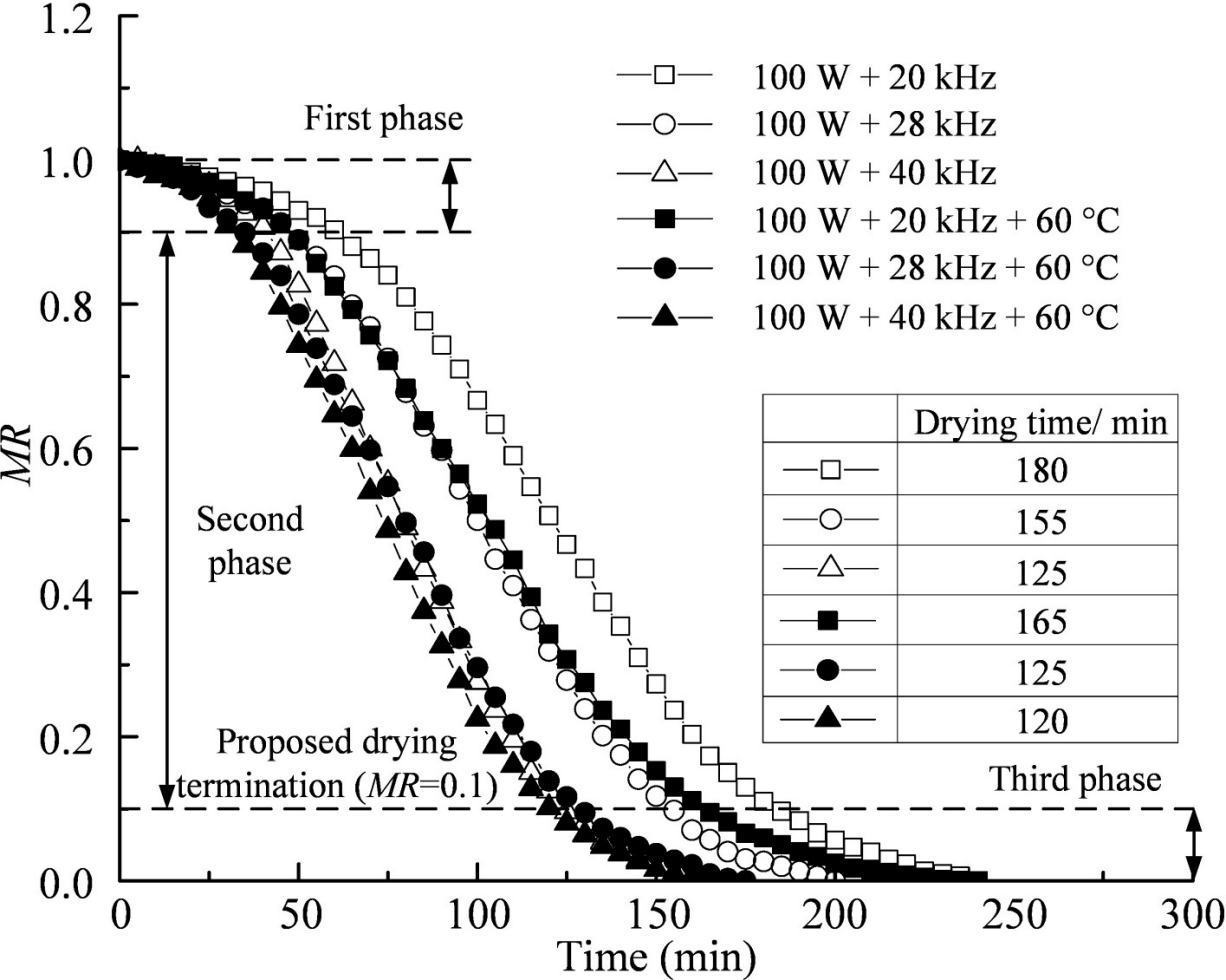
Variations in moisture ratio with time under the action of ultrasound at different frequencies without and with heating at 60°C.

The black and white symbols in Fig 5 represent the results of drying tests under the action of ultrasound with and without heating at 60°C, respectively. The power of the applied ultrasound was 100 W, and the frequencies were 20, 28 and 40 kHz. From the curves with white symbols, it can be seen that the drying rate without heating increases uniformly with the increase in ultrasound frequency. The length of drying time for the test with an ultrasound frequency of 40 kHz is 30.56% and 19.35% shorter than those with 20 kHz and 28 kHz, respectively. This phenomenon is attributed to the cavitation energy of ultrasound, which increases with the increase in frequency. Spongy effect produced by ultrasound in the silt also contributes to passage of water through pore channels [12].

From the curves with black and white symbols, it can be seen that the length of drying time under thermal drying combined with ultrasound was reduced by 8.33% and 19.35% of those under the action of ultrasound at frequencies of 20 and 28 kHz, respectively. However, the drying curves and the total lengths of drying time are approximately identical for the drying processes under 100 W + 40 kHz + 60°C and 100 W + 40 kHz. This implies that 40 kHz is almost the upper bound value of the frequency of ultrasound that is effective in expediting the thermal drying process.

### Thermal drying combined with ultrasound

**Fig 6:**
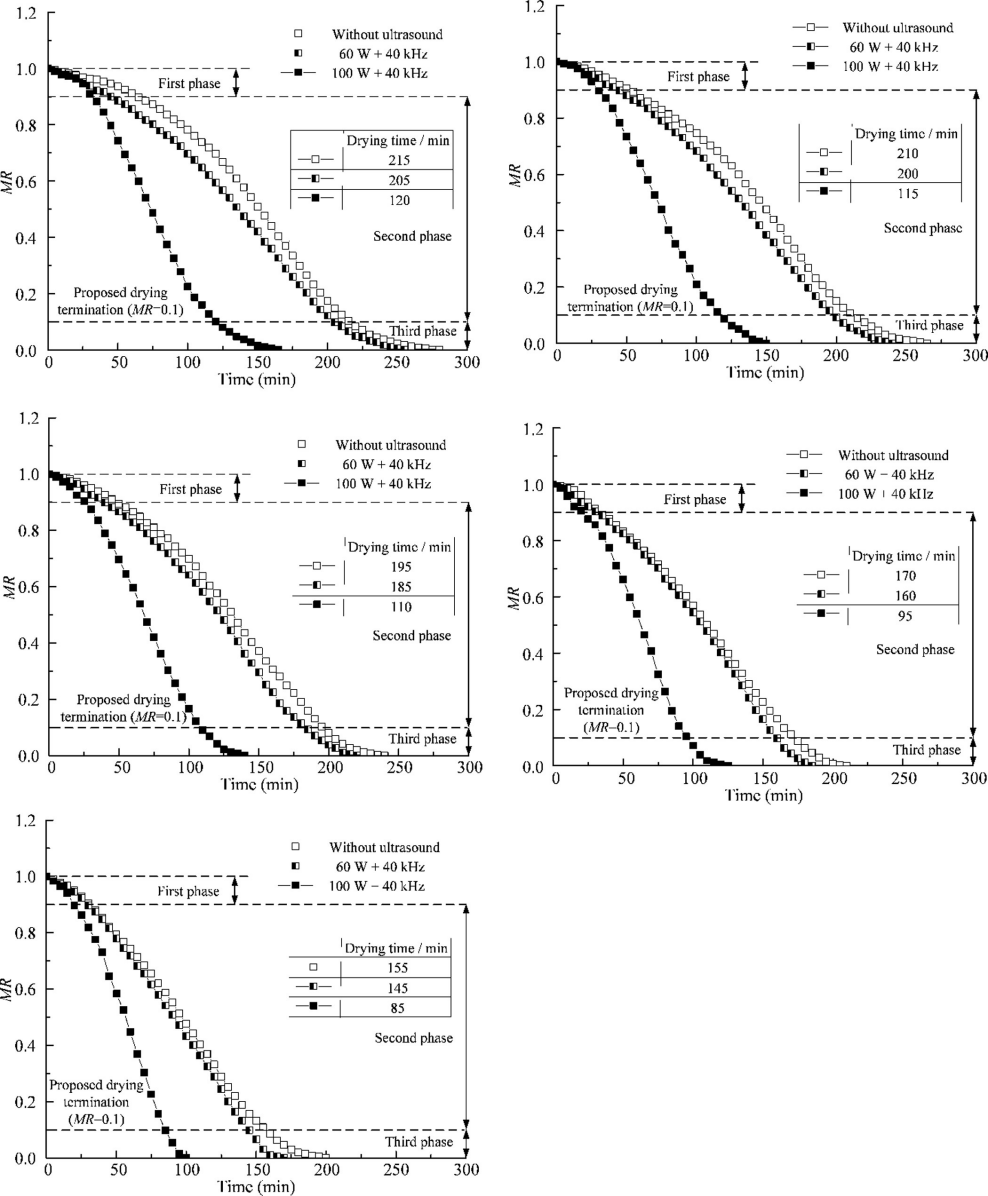
Influence of ultrasound power on thermal drying at different temperatures. (a) 60°C (b) 70°C (c) 80°C (d) 90°C (e) 100°C.

Fig 6(A)–6(E) show the results of the drying tests at different temperatures with and without ultrasound at a frequency of 40 kHz. The curves with white symbols represent the measured data for thermal drying. The curves with half–black symbols and black symbols represent the data of thermal drying with ultrasound powers of 60 W and 100 W, respectively. It can be seen that the drying rate increases with the increase in temperature. The application of ultrasound with power of 100 W functions better than that of 60 W in improving the drying rate of thermal drying. At the same temperature, the curve of thermal drying is close to the curve of thermal drying with 60 W ultrasound, but far away from the curve of thermal drying with 100 W ultrasound. This indicates that the application of ultrasound of low power has a limited effect on accelerating the thermal drying process.

Ultrasound of high power can increase the energy density per unit volume and improve the effect of cavitation and micro jet [28], generating micro-channels and pore structures in the silt. Thermal effect of ultrasound can also increase the temperature of specimen, improve the rate of water diffusion, and shorten the drying time. Fig 6(A)–6(E) clearly demonstrate that the drying time for thermal drying with ultrasound was significantly reduced as compared with thermal drying without ultrasound. When the temperature rises from 60°C to 70°C, 80°C, 90°C and 100°C, thermal drying combined with 100 W ultrasound can shorten the drying time by 44.19%, 45.24%, 43.59%, 44.12% and 45.16%, respectively; however, thermal drying combined with 60 W ultrasound can only shorten the drying time by 4.65%, 4.76%, 5.13%, 5.88% and 6.45%, respectively. The influence of ultrasound power on the drying time may be explained by the cavitation and thermal effects. The cavitation and thermal effects could contribute to water migration in the solid matrix and removal of the bound moisture. Therefore, the shortest drying time can be obtained by adopting thermal drying of 100°C combined with ultrasound of 100 W + 40 kHz.

The experimental data was also analyzed by applying analysis of variance (ANOVA). The factors considered in the analysis are the temperatures (60, 70, 80, 90 and 100°C) and ultrasound powers (0 W, 60 W and 100W). The significance levels of the ultrasound powers and temperatures were evaluated by their *F* values and *p* values, as presented in Table 2. From the *p* values, it can be seen that both temperature and ultrasound power have significant influence (*p* < 0.05) on the drying time of the silt. The ultrasound introduces pressure changes and microstreaming at the gas–solid interface. This leads to the reduction of the boundary layer thickness, and thus improves moisture transfer from interior to surface [29]. So, the *p* value of ultrasound is smaller than that of temperature, and the *F* value of ultrasound power is greater than that of temperature. Therefore, the drying time could be reduced by the application of ultrasound. The effect of ultrasound power on shortening the drying time is more significant than that of temperature.

**Table 2:** Analysis of variance for drying time.

Source	SS	DF	MS	*F* value	*p* value
Temperature	7614.65	4	1903.66	12.52	4.43×10^−4^
Ultrasound power	23761.11	2	11880.56	78.151	3.15×10^−7^
Error	1672.22	11	152.02		
Total	33047.98	17			

### Energy consumption and optimal scheme

In order to choose an optimum scheme of drying, it is necessary to consider its energy consumption. The energy consumption is composed of both the parts of heating and ultrasound. The heating equipment provides different temperatures corresponding to different powers: 60°C (400 W), 70°C (500 W), 80°C (600 W), 90°C (700 W) and 100°C (800 W).

**Fig 7:**
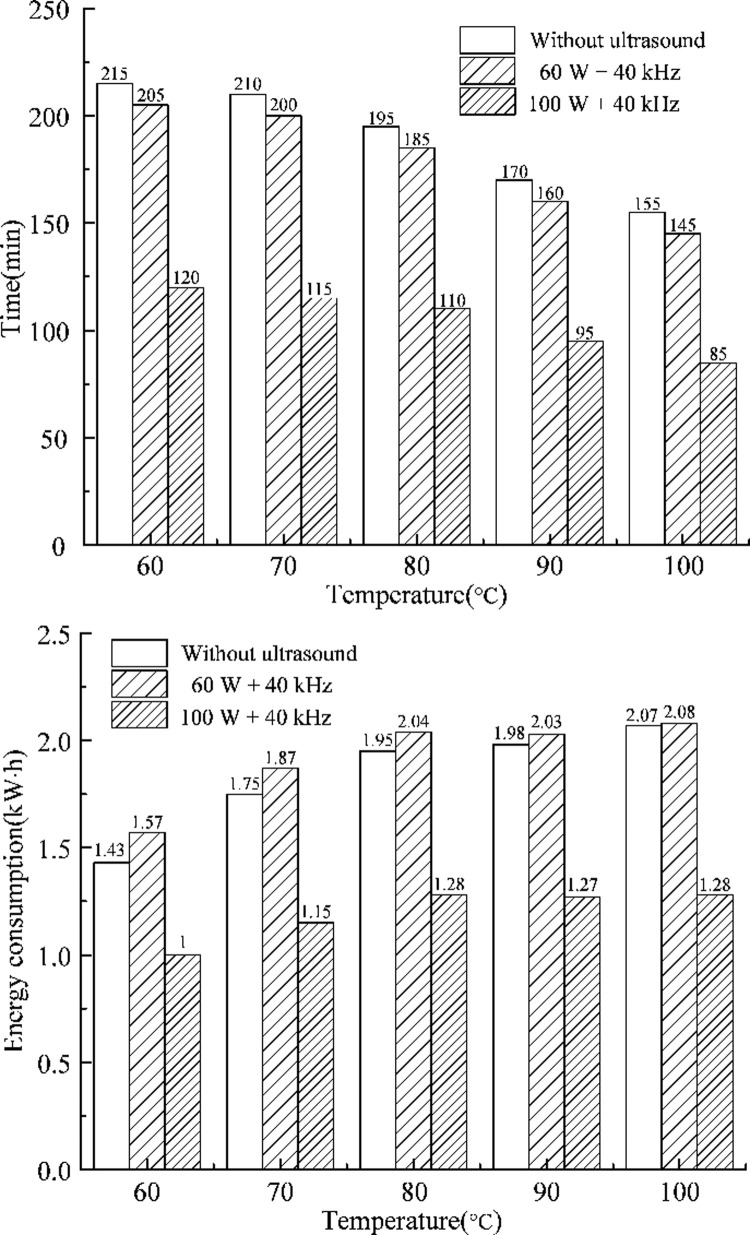
Drying time and energy consumption. (a) Drying time (b) Energy consumption.

The total energy consumption is equal to equipment power multiplied by drying time. The drying time and energy consumption versus temperature are shown in Fig 7(A) and 7(B), respectively. The drying time decreases with the increase in temperature, but the energy consumption continues to increase because the power of heating device increases with temperature. Compared with thermal drying, the energy consumption of thermal drying combined with 60 W ultrasound increased by 9.79%, 6.86%, 4.41%, 2.25% and 0.48% at different temperatures 60°C, 70°C, 80°C, 90°C and 100°C, respectively. In contrast, thermal drying combined with 100 W ultrasound can reduce the energy consumption by 30.07%, 34.29%, 34.36%, 35.86% and 38.16% at different temperatures 60°C, 70°C, 80°C, 90°C and 100°C, respectively. The reduction in the drying time leads to the decrease in the total energy consumption of the heating equipment as compared with thermal drying. However, the ultrasound equipment increases extra energy consumption. Therefore, the total energy consumption will be reduced only when the energy reduction of the heating equipment is greater than the energy consumption of the ultrasound equipment. In the first case, thermal drying assisted by ultrasound reduced little drying time than thermal drying. The energy consumption of ultrasound equipment is larger than the energy reduction of the heating equipment. Instead, in the second case, a significant reduction in the length of drying time caused that the energy consumption of ultrasound equipment is much smaller than the energy reduction of the heating equipment, especially when temperature reaches 80°C, at which the energy consumption did not increase. As a result, the total energy consumption of the first case increases and that of the second case decreases. Therefore, the length of drying time affects the energy consumption more significantly than the level of equipment power.

As shown in Fig 7(A) and 7(B), thermal drying (100°C) assisted by 100 W ultrasound costs the minimum drying time. The least energy consumption occurs in the case of thermal drying (60°C) assisted by 100 W ultrasound. Both drying time and energy consumption need to be considered to determine the optimal scheme. Thermal drying (100°C) assisted by 100W ultrasound costs more than 28% of energy and shortens 29.17% of drying time than thermal drying (60°C) assisted by 100 W ultrasound.

Results of the ANOVA are summarized in Table 3. It can be seen from the *p* values that both the temperature and ultrasound power have significant effects (*p* < 0.05) on the energy consumption. Table 3 shows that the *p* value of ultrasound is smaller than that of temperature, and the *F* value of ultrasound power is greater than that of temperature. This indicates that the ultrasound power has a more significant impact on the energy consumption than the temperature. Based on the above analyses, thermal drying (100°C) combined with 100W ultrasound is considered the optimal scheme for silt drying.

**Table 3:** Analysis of variance for energy consumption.

Source	SS	DF	MS	*F* value	*p* value
Temperature	0.46	4	0.12	23.5	1.77×10^−4^
Ultrasound power	1.56	2	0.78	160.06	3.53×10^−7^
Error	0.04	8	0.02		
Total	2.06	14			

## Conclusions

In this study, drying experiments of different modes were conducted to solve the problems existing in the process of harmless treatment and recycling of silt. Based on the experimental data, an analysis of variance was carried out to analyze the effects of temperature and ultrasound on drying time and energy consumption. The main conclusions are as follows:

1. In the cases of thermal drying without ultrasound, the length of drying time is shortened by 27.91% as the temperature increases from 60°C to 100°C.
2. The cavitation energy of ultrasound can expedite the drying of silt. In the cases of ultrasound drying with different frequencies, ultrasound with high frequency produces more cavitation energy than ultrasound with low frequency. The length of drying time for the case of ultrasound frequency at 40 kHz is 30.56% and 19.35% shorter than those at 20 kHz and 28 kHz.
3. Temperature and ultrasound are the significant factors influencing the drying time because the significance level is less than 0.05. Thermal drying combined with 100 W ultrasound can shorten drying time by 44.19%, 45.24%, 43.59%, 44.12% and 45.16% at temperatures 60°C, 70°C, 80°C, 90°C and 100°C, respectively. Thermal drying combined with 60 W ultrasound can only shorten drying time by 4.65%, 4.76%, 5.13%, 5.88% and 6.45% at temperatures 60°C, 70°C, 80°C, 90°C and 100°C, respectively.
4. The length of drying time affects the total energy consumption more significantly than the level of equipment power. The total energy consumption of the thermal drying combined with 60 W ultrasound increases, and that of the thermal drying combined with 100 W ultrasound decreases. At temperatures 60°C, 70°C, 80°C, 90°C and 100°C, the energy consumption of thermal drying combined with 60 W ultrasound was increased by 9.79%, 6.86%, 4.41%, 2.25% and 0.48%, and the energy consumption of thermal drying combined with 100 W ultrasound was reduced by 30.07%, 34.29%, 34.36%, 35.86% and 38.16%, respectively. In terms of drying time and energy consumption for silt drying, the combination of thermal drying with 100W ultrasound is found to be the optimal scheme.

## Supporting information

S1 File Date in Fig 2. (XLSX)

S2 File Date in Fig 4. (XLSX)

S3 File Date in Fig 5. (XLSX)

S4 File Date in Fig 6. (a) 60°C (b) 70°C (c) 80°C (d) 90°C (e) 100°C.
(RAR)
